# Blood-Brain Barrier Dysfunction Precedes Cognitive Decline and Neurodegeneration in Diabetic Insulin Resistant Mouse Model: An Implication for Causal Link

**DOI:** 10.3389/fnagi.2017.00399

**Published:** 2017-12-01

**Authors:** Ryusuke Takechi, Virginie Lam, Emily Brook, Corey Giles, Nicholas Fimognari, Armin Mooranian, Hani Al-Salami, Stephanie H. Coulson, Michael Nesbit, John C. L. Mamo

**Affiliations:** ^1^Curtin Health Innovation Research Institute, Curtin University, Perth, WA, Australia; ^2^School of Public Health, Faculty of Health Sciences, Curtin University, Perth, WA, Australia; ^3^School of Biomedical Sciences, Faculty of Health Sciences, Curtin University, Perth, WA, Australia; ^4^School of Pharmacy, Faculty of Health Sciences, Curtin University, Perth, WA, Australia

**Keywords:** blood-brain barrier, cognitive impairment, insulin resistance

## Abstract

Diabetic insulin resistance and pro-diabetic diet are reported to increase dementia risk through unknown mechanisms. Emerging evidence suggests that the integrity of blood-brain barrier (BBB) is central to the onset and progression of neurodegeneration and cognitive impairment. Therefore, the current study investigated the effect of pro-diabetic diets on cognitive dysfunction in association to BBB integrity and its putative mechanisms. In C57BL/6J mice chronically ingested with a diet enriched in fat and fructose (HFF), Morris Water Maze (MWM) test indicated no significant cognitive decline after 4 weeks of HFF feeding compared to low-fat (LF) fed control. However, at this stage, BBB dysfunction accompanied by heightened neuroinflammation in cortex and hippocampal regions was already evident. After 24 weeks, HFF fed mice showed significantly deteriorated cognitive function concomitant with substantial neurodegeneration, which both showed significant associations with increased BBB permeability. In addition, the data indicated that the loss of BBB tight junctions was significantly associated with heightened inflammation and leukocyte infiltration. The data collectively suggest that in mice maintained on pro-diabetic diet, the dysfunctional BBB associated to inflammation and leukocyte recruitment precedes the neurodegeneration and cognitive decline, possibly indicating causal association.

## Introduction

Population studies report that type-2 diabetes (T2DM) significantly increases the risk of dementia by ~5-fold and indeed diabetic insulin resistance is an established risk factor for Alzheimer’s disease (AD; Craft et al., 2000; Bowman et al., 2007, 2012; Barbagallo and Dominguez, 2014; Simó et al., 2017). Rotterdam Study revealed that T2DM increases the risk of AD by 1.9-fold (95%CI: 1.2–3.1; Ott et al., 1999). In a cross-sectional study by Ohara et al. (2011), a relative risk of AD was 2.05 (95%CI: 1.18–3.57), while a relative risk for vascular dementia was 1.82 (95%CI: 0.89–3.71). Preclinical studies in rodents with dietary-induced or genetic insulin resistance also show significant cognitive impairment generally associated with the severity and duration of diabetes (Kanoski and Davidson, 2010; McNeilly et al., 2012). Purported causal mechanisms may include altered glycolysis (Zheng et al., 2017) and depressed neuronal mitochondrial activity, attenuated BDNF, insulin desensitization (also known as type-3 diabetes) possibly as a consequence of depressed adiponectin (Ng et al., 2016), exaggerated BACE1 mediated amyloid biogenesis (Lee et al., 2016), and tau phosphorylation (Platt et al., 2016). General consensus from cross-sectional studies is that cognitive impairment is profoundly associated and induced by hyperglycemia. However, recent longitudinal studies report conflicting results showing no agreements between the onset of AD and glucose tolerance (Kawamura et al., 2012). In concert with this disputing finding, McNeilly et al. (2012) reported that an improvement of insulin sensitivity by metformin did not ameliorate cognitive dysfunction in a dietary-induced rat model of T2DM. While emerging evidence suggest that dysregulation of neuronal insulin signaling, so-called cerebral insulin resistance, may play central role in cognitive dysfunction (de la Monte, 2012; Ferreira et al., 2014; De Felice and Benedict, 2015; Grillo et al., 2015; Nuzzo et al., 2015), the exact mechanisms by which T2DM influences the cognitive function and dementia risk are yet to be elucidated.

An emerging body of evidence suggests that compromised integrity of cerebrovascular blood-brain barrier (BBB) precedes cognitive decline in AD, indicating its causal association (Bell and Zlokovic, 2009; Stolp and Dziegielewska, 2009; Zlokovic, 2011). Ordinarily, cerebral neuronal function and signaling are protected from blood-borne potentially neurotoxic macromolecules as a consequence of the restricted transport and otherwise impermeable properties of the BBB. Functional disturbances in cerebrocapillaries are broadly characterized by altered permeability, cerebral extravasation of plasma molecules, neuroinflammatory and oxidative sequalae, and thereafter, progressive loss of neuronal function and neuronal apoptosis (Ramirez et al., 2009). These features are consistently indicated in T2DM and provide mechanistic insight as to how T2DM might heighten the risk for AD via a cerebrovascular axis. However, there is a limited number of studies reporting the effects of diabetic insulin resistance on cerebral capillary integrity, and indeed none directly exploring the putative association of BBB dysfunction with cognitive loss (Huber, 2008). Starr et al. (2003) showed significantly greater BBB permeability in subjects with well-controlled T2DM. In leptin receptor deficient insulin resistant mice, Stranahan et al. (2016) showed that BBB breakdown results in cerebral macrophage infiltration via an IL-1β facilitated pathway. In F1 progeny of amyloid transgenic APP/PS1 mice crossed with diabetic db/db mice, Ramos-Rodriguez et al. (2015) reported that BBB alterations occurred concomitant with the onset of T2DM (14 weeks) and preceded amyloidosis. However, in the latter study, there were no synergistic effects with hallmark pathologies at later age (26 weeks). Findings from the Maastricht Study recently demonstrated that prediabetes, T2DM and measures of hyperglycemia were independently associated with impaired microvascular function in the retina and skin (Sörensen et al., 2016). The authors concluded that microvascular dysfunction precedes and thus may contribute to T2DM-associated complications such as impaired cognition. However, Jansen et al. (2016) in 41 T2DM patients found no evidence of cerebral hypoperfusion with cognitive decline, ruling out frank capillary collapse as a potential vascular mediated regulator of cognitive performance.

In light of the clinical and preclinical studies indicated, the purpose of this study was to directly explore the hypothesis that significant aberrations in BBB permeability associates with cognitive function in pre-diabetic insulin resistant mouse model. A commonly utilized, clinically relevant dietary fat and sugar induced wild-type mouse model of pre-diabetic insulin resistance was adopted to avoid the potential confounder of severe, poorly controlled T2DM commonly indicated in genetic strains of mice.

## Materials and Methods

### Ethical Approval

All animal procedures were approved by Curtin Animal Ethics Committee (approval no. AEC_2013_23) and performed in accordance with Australian Code for the Care and Use of Animals for Scientific Purposes 8th Edition.

### Animals and Dietary Intervention

Male 6-week old C57BL6/J mice were purchased from Animal Resources Centre (WA, Australia). Mice were randomly allocated to either control or diabetic group (*n* = 20). The sample size was determined based on previous studies (Al-Salami et al., 2017; Mamo et al., 2017). Mice in control group received standard low-fat (LF) chow (AIN-93M, Specialty Feeds, WA, Australia), while diabetic group received a pro-diabetic diet containing high fat and fructose (HFF; 30% (w/w) lard, 0.5% (w/w) cholesterol and 15% (w/w) fructose (SF14-088, see Supplementary Tables S1, S2 for detail). The intervention duration of 4 weeks was chosen based on our pilot data, which indicated the onset of mild-insulin resistance, while 24 weeks was to explore the chronic effects of insulin resistance on cognitive function and BBB permeability. The mice of both LF and HFF group had *ad libitum* access to the chow and water.

### Measurement of Blood Glucose, HbA1c, Plasma Insulin and Homeostasis Model Assessment IR

Non-fasting blood glucose was measured immediately before the sacrifice of the mice via tail vein puncture using AccuCheck Go glucometer (Roche Laboratories, Switzerland). HbA1c level was determined with Siemens DCA Vantage Analyzer with DCA HbA1c reagent cartilage (Siemens Healthcare Diagnostics, USA). Plasma insulin concentration was determined by commercial ELISA kit (Mercodia, Sweden) according to the manufacturer’s instruction. In order to assess the degree of insulin resistance, Homeostatic Model Assessment—Insulin Resistance (HOMA-IR) was calculated based on the blood glucose and insulin levels by using HOMA Calculator version 2.2.3 (Diabetes Trials Unit).

### Assessment of Cognitive Function with Morris Water Maze

After 4 and 24 weeks of intervention with pro-diabetic diet, cognitive performance was determined utilizing Morris Water Maze (MWM), considered an appropriate tool for spatial learning and short-term working memory as described previously (Ueno et al., 2002; Bélanger et al., 2004). Briefly, a white colored pool (120 cm diameter × 60 cm height) was used. The pool was filled with water to 30 cm height. A clear colored platform (10 cm diameter) was placed in the N quadrant of the pool. The mice received training for 2 days with a visible platform (1 cm above the water surface) without visual cues. Each day consisted of four trials. Each mouse was allowed to search the platform for a maximum of 90 s per trial. After reaching the platform, the mouse was left for 30 s on the platform. In order to minimize the variance of mental and physical status such as stress, motivation and locomotor function, the mice that did not reach the platform within 10 s by the end of the training were excluded. After this exclusion, all groups had at least six mice for further analyses.

The actual maze test was performed with a submerged invisible platform (1 cm below the water surface) over five consecutive days and with distinct visual cues set up in each N, S, E and W quadrant. Each day consisted of four trials and each trial was for 90 s with 30 s on platform. Latency to reach the platform and swim speed were recorded with HVS Image (Buckingham, UK). On the following day of test trial Day 5, a probe trial was performed to assess long-term memory recall. A probe trial consisted of only one 60 s trial by removing the escape platform. The time that mouse spent in a correct quadrant of the pool was recorded.

### Assessment of BBB Integrity with 3-D Semi-Quantitative Microscopy and Flow Cytometry

As previously demonstrated by our laboratory and others, cerebral parenchymal extravasation of IgG was semi-quantitatively measured as a marker of BBB permeability by using immunofluorescent confocal microscopy imaging in a blinded manner (Takechi et al., 2010, 2013b,c). Briefly, the right brain hemisphere was fixed in 4% paraformaldehyde for 24 h followed by cryoprotection with 20% sucrose for 72 h. Twenty micrometer thick cryosections were incubated with Alexa488 conjugated with rabbit anti-mouse IgG (1:100, Life Technologies, CA, USA) for 20 h at 4°C. 3-D confocal immunofluorescent images were captured with UltraView Vox microscopy (PerkinElmer, MA, USA) at 200× magnification, and each 3-D image consisted of 21 2-D images (1000 × 1000 pixels, 346 μm × 346 μm, 1 μm z-axis distance). An average of 20 and 10 images were randomly captured in the regions of cortex and hippocampal formation (HPF), respectively. Voxel intensity of Alexa488 fluorescence in the cerebral parenchyme was determined with Volocity (ver 5.4, PerkinElmer). In order to exclude intravascular IgG, blood vessels were identified by automatic object selection module of Volocity by using intensity threshold, which followed by investigator’s manual confirmation based on the DAPI nuclei staining. Mean voxel intensity was calculated per volume unit per animal, then the group mean was calculated.

In order to confirm the data of BBB permeability assessed by the parenchymal extravasation of IgG, the expression of BBB endothelial tight junction proteins, occludin-1 and ZO-1, was semi-quantitatively determined with flow cytometry as established previously in our laboratory (Elahy et al., 2015). Briefly, fresh left brain hemisphere was sliced in FACs buffer with 10 μg/ml brefeldin (Sigma-Aldrich, St. Louis, MO, USA). The tissue slice was then digested in FACs buffer with 1 mg/ml collagenase IV (Sigma-Aldrich), 1 mg/ml dispase (Sigma-Aldrich), 1 mg/ml DNase (Sigma-Aldrich) and 2.5 μg/ml brefeldin at 37°C for 30 min. The obtained single cell suspensions were incubated with fluorescently labeled antibodies against extracellular markers of anti-CD31-BV421 (1:200, Biolegend, CA, USA) and anti-CD45-PerCP-Cy5.5 (1:500, Biolegend). For intracellular marker staining, cells were permeabilized with 0.1% saponin (Sigma-Aldrich) for 15 min and incubated with rabbit anti-mouse occludin-1 (1:50, Abcam, UK), or rabbit anti-mouse ZO-1 (1:100, Abcam) for 30 min at 4°C followed by an incubation with goat anti-rabbit IgG Alexa488 (1:500, LifeTechnologies) for 30 min at 4°C. Samples were then acquired on FACS Canto II (BD Biosciences, NJ, USA). Cerebrovascular endothelial cells (ECs) were gated as CD31 positive CD45 negative (see Figure 3D and Supplementary Figure S1 for example gating strategy). In order to evaluate the protein expression of interest per EC, the mean fluorescent intensity of occludin-1 and ZO-1 was analyzed with FlowJo V10 software (Treestar, OR, USA), and expressed as per EC.

**Figure 1:**
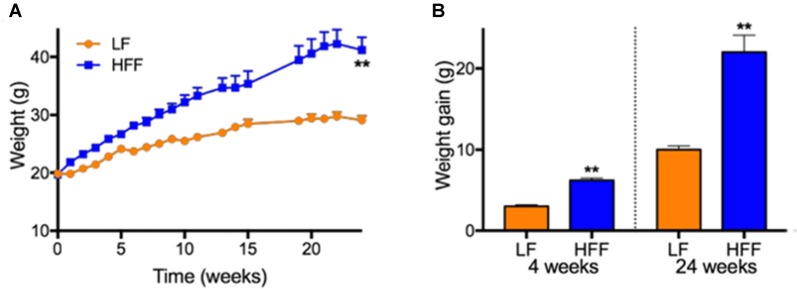
Weights and weight gain. **(A)** Weights of mice maintained on control low-fat (LF) and high fat/fructose (HFF) diet for 4 and 24 weeks are indicated. **(B)** Mean weight gains of individual mouse during 0–4 weeks and 0–24 weeks are indicated. Statistical significance between LF and HFF was tested with *t*-test and indicated with asterisk (**p < 0.001; *n* = 20 between 0–4 weeks; *n* = 10 between 5–24 weeks).

**Figure 2:**
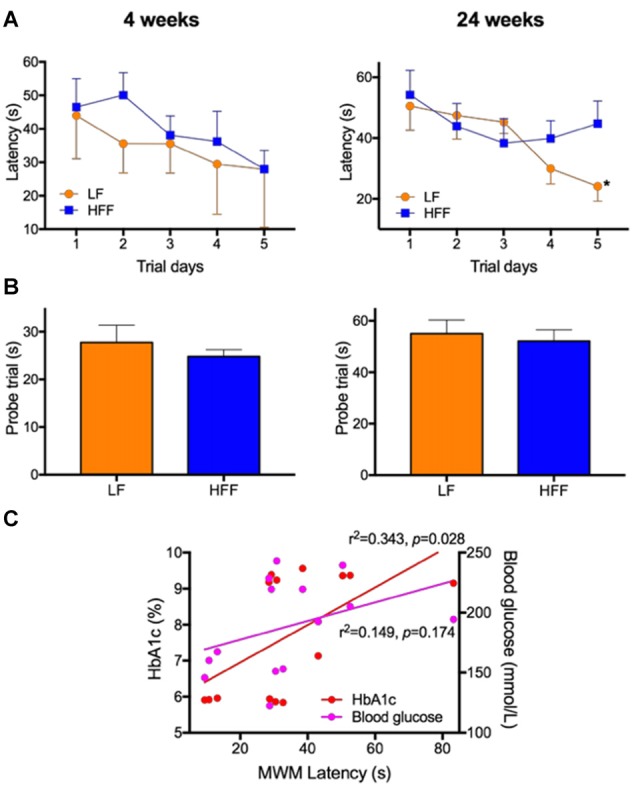
Assessment of cognitive function with Morris Water Maze (MWM). MWM was used to assess cognitive function of mice maintained on LF control or HFF diet for 4 and 24 weeks. **(A)** Latency to reach the platform was indicated from Day 1 to 5 of the trials, showing spatial learning and memory. Statistical significance between LF and HFF was tested by *t*-test and indicated with asterisk (*p < 0.05; *n* = 10). **(B)** Long-term reference memory was tested by probe trial and the time spent in the correct quadrant is presented. **(C)** The association of insulin resistance with cognitive decline was considered by correlation coefficient analysis between the MWM Day 5 latency vs. plasma insulin and HbA1c.

**Figure 3:**
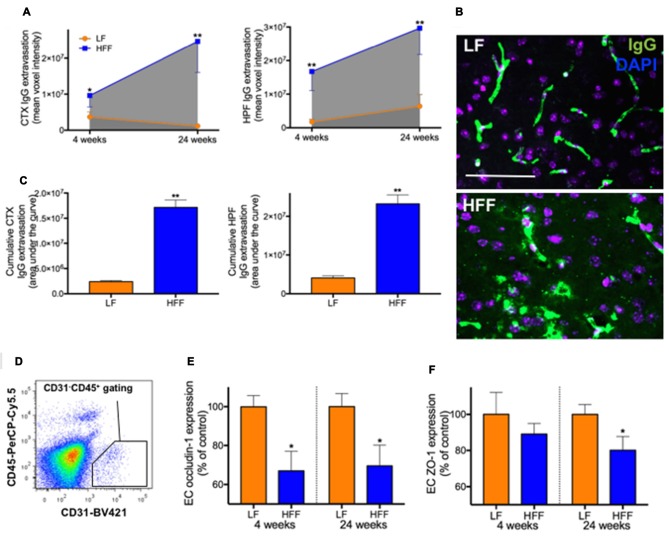
Integrity of blood-brain barrier (BBB) assessed by immunomicroscopy and flow cytometry. The cerebral parenchymal extravasation of IgG was semi-quantitatively measured by confocal immunomicroscopy as a surrogate marker of BBB integrity in mice maintained on LF and HFF diet for 4 and 24 weeks. **(A)** IgG extravasation was measured in the cortex (CTX) and hippocampal formation (HPF). **(B)** Representative immunomicroscopy images from the cortex of mice fed with LF and HFF diet for 24 weeks are shown (green: IgG; purple: DAPI; scale bar = 50 μm). **(C)** Area under the curve of parenchymal IgG extravasation was calculated from **(A)**, showing cumulative effects of BBB disruption over the entire experimental period. **(D)** The diagram shows an example of gating strategy for cerebrovascular endothelial cells (ECs) used for flow cytometry analysis of BBB tight junction protein expression (CD31 positive CD45 negative). The EC expression of tight junction proteins, occludin-1 **(E)** and ZO-1 **(F)**, are indicated as percent of LF control. Statistical significance between LF and HFF was tested by *t*-test and indicated with asterisk (*p < 0.05; **p < 0.001; *n* = 10).

### Semi-Quantitative Measurement of Astrocyte Activation and Neurodegeneration

Double staining 3-D semi-quantitative microscopy was used to determine cerebral astrocyte activation and neurodegeneration by using GFAP and Fluoro-Jade C as described previously (Bian et al., 2007; Takechi et al., 2013b,c). Briefly, 20 μm cryosections were incubated with rabbit anti-mouse GFAP (1:200, Abcam) for 20 h at 4°C. The sections were then incubated with goat anti-rabbit IgG conjugated with Alexa488 for 2 h at 20°C. After the sections were dried for 10 min at 60°C, Fluoro-Jade C (Biosensis, SA, Australia) staining was done. The sections were incubated with sodium hydroxide (Solution A) for 5 min. Subsequently the sections were incubated with potassium permanganate (Solution B) for 10 min. Finally, the sections were incubated with Fluoro-Jade C (Solution C) and DAPI (Solution D) for 10 min in dark. Confocal microscopy imaging and the measurement of voxel fluorescent intensity of GFAP and Fluoro-Jade C were done as described above.

### Determination of BBB Endothelial Inflammation, Oxidative Stress and Monocyte Recruitment

Surrogate markers of inflammation, oxidative stress and monocyte recruitment of BBB ECs were determined by using flow cytometry as established previously (Elahy et al., 2015). Briefly, the single cell suspensions were incubated with extracellular markers of anti-CD31-BV421 (1:200), anti-CD45-PerCP-Cy5.5 (1:500), anti-VCAM-1-APC (1:200, Biolegend) and anti-ICAM-1-PE (1:200, Biolegend) for 30 min at 4°C. Following the permeabilization, the cells were incubated with anti-TNF-α-APC (1:50, biolegend), anti-IL-1β-FITC (1:50, eBioscience, CA, USA) and dihydroethidium (DHE, 3.0 μg/L, Sigma-Aldrich) for 30 min at 4°C. Samples were acquired through FACS Canto II with a gating for CD31^pos^CD45^neg^. The expression of each protein of interest was determined by calculating the fluorescent intensity per EC.

### Statistical Analyses

To obtain sufficient statistical power to detect the effects of HFF diet on BBB integrity and cognitive behavior, 10 mice were used per duration per group based on previous studies. All data is presented as mean ± SEM. As a marker of cumulative effects over the 24-week dietary intervention, AUC was estimated (Prism 7, GraphPad, CA, USA). The normality of Gaussian data distribution was assessed by D’Agostino-Pearson normality test. For the data that were normally distributed, the statistical differences between LF and HFF mice were assessed by parametric unpaired *t*-test (two-tailed), while the data that were not normally distributed were analyzed by nonparametric Mann-Whitney test (two-tailed). Associations were analyzed by pooling the data from LF and HFF groups with linear regression analysis. In addition, correlation coefficient was analyzed with Pearson’s correlation coefficient and Spearman’s correlation coefficient analyses for the data that were normally and not normally distributed, respectively. Statistical significance was indicated at *p* < 0.05.

## Results

### HFF Feeding Induced Mild- and Moderate-Insulin Resistance after 4 and 24 Weeks, Respectively

Mice maintained on HFF diet for 4 and 24 weeks had significantly higher weight gain compared to mice receiving control LF chow (Figure 1). Blood glucose and HbA1c of HFF mice were significantly higher than LF mice at 4 weeks and 24 weeks after the commencement of dietary intervention (Table 1). Following 24 weeks of HFF feeding, blood glucose and HbA1c were 224.3 ± 6.4 mg/dL and 9.35 ± 0.04% (78.7 ± 0.5 mmol/mL) respectively, indicating pre-diabetic state. Progressive insulin resistance was also suggested based on a modest increase of plasma insulin at 4 weeks of intervention vs. a 5-fold elevation to 1.09 ± 0.283 ng/mL at 24 weeks of HFF-feeding (Table 1). Consistently, HOMA-IR of mice maintained on HFF diet for 4 weeks was 19.2 ± 5.3, which was significantly greater compared to LF control mice. After 24 weeks, HOMA-IR significantly increased to 39.6 ± 9.6 in HFF mice, indicating progressive insulin resistance.

**Table 1:** Blood glucose, HbA1c, plasma insulin and HOMA-IR.

	4 weeks		24 weeks	
	LF	HFF	LF	HFF
Glucose (mg/dL)	99.7 ± 4.1	196.9 ± 6.1**	157.7 ± 6.1	224.3 ± 6.4**
HbA1c (%)	5.12 ± 0.08	8.38 ± 0.18**	5.92 ± 0.12	9.35 ± 0.04**
(mmol/mol)	(32.5 ± 0.9)	(68.1 ± 2.0**)	(42.3 ± 1.3)	(78.7 ± 0.5**)
Insulin (ng/mL)	0.24 ± 0.087	0.51 ± 0.155*	0.19 ± 0.041	1.09 ± 0.283**
HOMA-IR	8.1 ± 2.7	19.2 ± 5.3**	7.2 ± 1.5	39.6 ± 9.6**

LF, low-fat fed control group; HFF, high fat and fructose fed group; HOMA-IR, Homeostatic Model Assessment Index—Insulin Resistance. *n* = 10; asterisk indicates statistical significance compared to LF by t-test (*p < 0.05, **p < 0.01).

### Cognitive Decline Was Not Detected after 4 Weeks, Which Became Evident after 24 Weeks

As presented in Supplementary Figure S2, there were no significant differences in the swim speed of mice during MWM between any intervention groups, indicating similar levels of locomotor function and motivation/mental status. After 4 weeks of dietary intervention, there were no significant differences in the MWM latency on trial Day 5 between LF control and HFF-fed mice, indicating no deterioration of spatial learning and memory (Figure 2A). A long-term spatial reference memory assessed by the probe trial showed no significant changes (Figure 2B). However, after 24 weeks of HFF feeding, Day 5 maze latency was significantly greater compared to LF control mice, indicating impaired spatial learning and memory (Figure 2A). Long-term memory recall was not affected by the 24-week HFF feeding (Figure 2B). A significant correlation coefficient was shown between the maze latency time with plasma insulin and HbA1c (Figure 2C).

### Substantial BBB Breakdown Observed after 4 Weeks, Which Further Progressed to 24 Weeks

Semi-quantitative immunomicroscopy analysis showed significantly elevated cerebral parenchymal extravasation of IgG in both cortex and HPF of mice maintained on HFF diet for 4 weeks compared to LF fed control mice (2-fold and 9-fold increase, respectively), indicating increased BBB permeability (Figures 3A–C). Flow cytometry analysis of cerebrovascular EC (Figure 3D) confirmed the dysfunction of BBB by showing significantly decreased endothelial expression of BBB tight junction protein, occludin-1 in HFF-fed mice at 4 weeks compared to control mice by 35% (Figure 3E), while no changes in EC numbers were observed (data not shown). After 24 weeks of dietary intervention, mice receiving HFF had substantially amplified BBB disruption in cortex and hippocampus (Figures 3A,B), which was supported by the flow cytometry data showing further attenuation occludin-1 and ZO-1 expression in the BBB endothelium of HFF fed mice compared to LF control mice (Figures 3E,F). The IgG extravasation AUC of HFF fed mice was more than 6-fold greater compared to control mice in both cortex and HPF (Figure 3C).

### Elevated Neuroinflammation and Neurodegeneration in HFF-Fed Mice Were Associated with BBB Dysfunction

**Figure 4:**
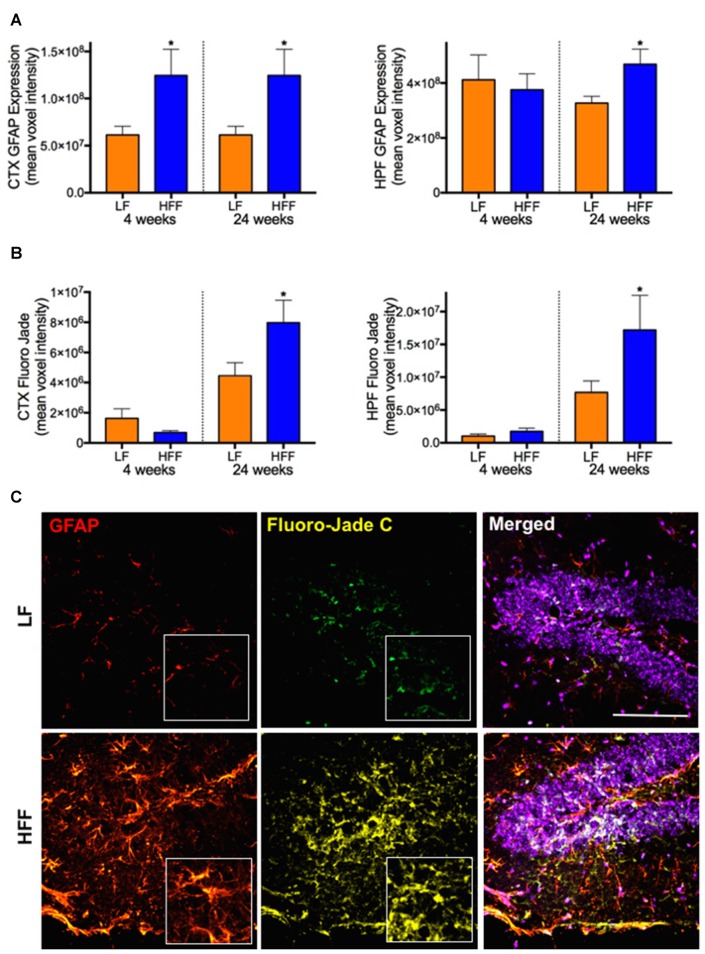
Markers of astrocyte activation and neurodegeneration. Activated astrocytes and neurodegeneration were assessed by semi-quantitative confocal immunomicroscopy analysis of GFAP and Fluoro-Jade C, respectively in the cortex (CTX) and hippocampus (HPF) of mice maintained on LF and HFF diet for 4 and 24 weeks. Mean fluorescent voxel intensities of GFAP and Fluoro-Jade are shown in frames **(A,B)**, respectively. Statistical significance between LF and HFF was tested by *t*-test and indicated with asterisk (*p < 0.05; *n* = 10). **(C)** Representative microscopic images at 24 weeks are shown from hippocampal region of LF and HFF mouse brains with 4× magnified inserts (red: GFAP; yellow: Fluoro-Jade C; purple: DAPI nuclei staining; scale bar = 50 μm).

Significant double-fold increase of astrogliosis and astrocytosis assessed by quantitative immunomicroscopic analysis of GFAP expression was demonstrated in the cortex after 4 weeks of HFF ingestion compared to LF (Figure 4A), indicating the evolution of astrocytic activation. Neurodegeneration measured by Fluoro-Jade C staining was not observed in HFF or LF mice at 4 weeks (Figure 4B). However, at 24 weeks of the dietary intervention, the mean fluorescent intensities of GFAP and Fluoro-Jade C staining were significantly elevated both in the cortex and HPF of HFF fed mice (Figures 4A–C).

**Figure 5:**
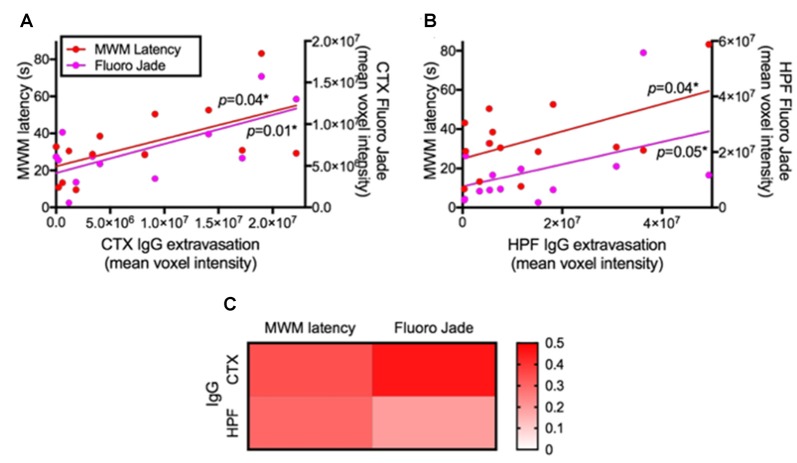
Association between BBB integrity and cognitive function/neurodegeneration. The associations of BBB integrity with cognitive function and neurodegeneration were considered by correlation coefficient analysis after 24 weeks of LF and HFF feeding. **(A)** shows correlation coefficient analysis between IgG extravasation in cortex (CTX) vs. Day 5 MWM latency and vs. Fluoro-Jade C in CTX, while **(B)** shows the analysis in the HPF. Significant correlation was assessed at *p* < 0.05. **(C)** R squared values of linear regression are shown as a heatmap.

The correlation coefficient analysis results show that increased permeability of BBB indicated by the parenchymal IgG extravasation was significantly associated with delayed maze latency and increased Fluoro-Jade C staining in both cortex (Figure 5A) and HPF (Figure 5B). A heatmap of linear regression r squared values indicates stronger associations in the cortex compared to hippocampus (Figure 5C).

### Loss of BBB Tight Junction Was Associated with Increased TNF-α and Adhesion Molecules

**Figure 6:**
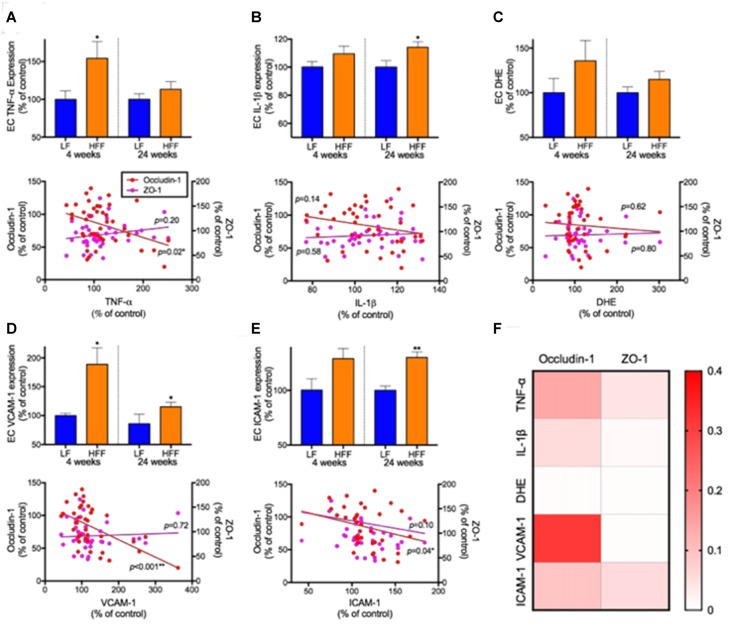
Markers of neurovascular inflammation, oxidative stress and leukocyte infiltration and their association with tight junction protein expression. The inflammation, oxidative stress and leukocyte infiltration in EC of cerebrovasculature were determined with flow cytometry. The EC inflammation was measured with the expression of TNF-a **(A)** and IL-1β **(B)**. The production of reactive oxygen species was measured by dihydroethidium (DHE; **C**). The leukocyte recruitment was assessed by the expression of adhesion molecules, VCAM-1 **(D)** and ICAM-1 **(E)**. The associations of neurovascular inflammation, oxidative stress and leukocyte infiltration with the expression of cerebrovascular EC expression of tight junction proteins were considered by correlation coefficient analysis. Significant association was assessed at *p < 0.05 and **p< 0.01. **(F)** R squared values of linear regression are shown as a heatmap.

The BBB EC expression of TNF-α was significantly elevated by 50% in HFF mice at 4 weeks compared to LF control (Figure 6A). Similarly, the expression of IL-1β showed significant increase by 15% in HFF fed mice at 24 weeks (Figure 6B). No statistical significance was detected in a marker of oxidative stress, DHE (Figure 6C). VCAM-1 expression, a surrogate marker of endothelial activation, was significantly elevated in HFF mice at both 4 weeks and 24 weeks of intervention (Figure 6D). In agreement with the latter, another marker of endothelial activation, ICAM-1, was significantly exaggerated after 24 weeks of HFF feeding (Figure 6E).

Correlation coefficient analysis indicated that BBB EC expression of occludin-1 was significantly negatively correlated with TNF-α. Significant negative correlations were also observed between endothelial occludin-1 vs. VCAM-1 and vs. ICAM-1. A heatmap summary of linear regression r squared values (Figure 6F) shows stronger associations with occludin-1 compared to ZO-1. Weak associations were found with DHE.

## Discussion

Diabetic insulin resistance is reported to accelerate cognitive decline and increase dementia risk, however the mechanisms for a causal association are presently not established. An accumulating body of recent studies suggest that integrity of BBB is pivotal to cognitive decline and dementia risk. Therefore, the primary aim of the present study was to investigate whether pre-diabetes might amplify cognitive decline concomitant with functional disturbances in BBB integrity. A widely utilized dietary-induced murine model of pre-diabetes was adopted so as to avoid significant diabetes that occurs in strains with genetic mutations (Calvo-Ochoa et al., 2014; Wooten et al., 2016). Whilst the indicated model is recognized as a physiologically relevant model of human pre-diabetic insulin resistance, dietary effects independent of insulin sensitivity must also be considered.

Animal studies reported markedly impaired cognitive function in insulin resistance. McNeilly et al. (2011) showed significant cognitive decline in dietary-induced insulin resistant rats indicated by lowered performance in switching task contingency of a delayed matching and non-matching to position task. Similarly, in a study with high fat-induced insulin resistance mice, animals performed poorly in Y-maze and MWM compared to healthy control mice (Jiang et al., 2012). However, to date, none of the studies indicated directly considered BBB integrity in association to cognitive function. In the present study, pre-diabetes was indicated by HOMA-IR as early as 4 weeks of intervention with greater plasma glucose HbA1C and insulin in HFF mice compared to LF controls. Following 24 weeks of dietary intervention, HOMA-IR, plasma glucose and HbA1c were significantly increased. Moreover, plasma insulin was significantly elevated to 0.51 ± 0.155 ng/ml, clearly indicating pre-diabetic insulin resistance as reported by other studies using similar diets (Tran et al., 2009; Calvo-Ochoa et al., 2014; Wooten et al., 2016).

By using this murine model of pre-diabetes, our results for the first time demonstrated that the breakdown of BBB integrity precedes the development of cognitive decline and neurodegeneration. At 4 weeks of dietary intervention suggesting the onset of pre-diabetes, HFF-fed mice were cognitively normal compared to the LF controls. In concert with this finding, no sign of exaggerated neurodegeneration indicated by Fluoro Jade-C staining was observed. However, the BBB permeability was significantly increased within 4 weeks of commencing a pro-diabetic diet, which was accompanied by the activation of astrocytes. Markedly compromised BBB integrity was indicated within both the cortex and HPF of HFF-fed mice while significant elevated neuroinflammation was concomitantly observed in the cortex. Following 24 weeks of dietary intervention suggesting pre-diabetic insulin resistance, the cognitive performance in HFF-fed mice became significantly impaired compared to healthy LF control mice. Concomitantly, significantly exaggerated neurodegeneration was observed within the cortex and hippocampal regions of HFF mice, while no sign of neuroinflammation and neurodegeneration was observed in LF control mice. Our findings of disrupted BBB preceding cognitive decline are coherent with some recent studies suggesting the pivotal involvement of BBB dysfunction during the onset and early progress of dementia and AD (van de Haar et al., 2016). This notion is further supported by our correlation analyses showing significant associations of BBB permeability with cognitive decline and with neurodegeneration.

The mechanisms by which pre-diabetes compromises BBB integrity and heightens neurodegeneration are not equivocal. A number of studies reported that heightened inflammation, oxidative stress, pericyte dysfunction and leukocyte recruitment result in the loss of BBB EC structure of tight junction complex (Kam et al., 2012; Shah et al., 2013). *In vitro* and *in vivo* studies reported that TNF-α increased BBB permeability by suppressing the expression of tight junction proteins (Mark and Miller, 1999; Trickler et al., 2005). In addition, TNF-α is reported to upregulate the expression of ICAM-1 and VCAM-1 in cerebral ECs (Ohara et al., 2000). Similarly, a study by Blamire et al. (2000) demonstrated that rats administered with IL-1β exhibit the dysfunction of BBB and neutrophil infiltration. A study by Stolp et al. (2011) reported that systemic inflammation induced by LPS injection results in increased permeability of BBB through the loss of tight junction expression and compromised behavior. In agreement with these reports, in the current study, all measures of cerebral EC inflammation and activation showed increasing trends in HFF-fed mice with statistically significant elevation in the EC expressions of TNF-α, IL-1β, VCAM-1 and ICAM-1. Moreover, correlation analyses showed that TNF-α, VCAM-1 and ICAM-1 are negatively associated with BBB EC expressions of occludin-1, while weaker associations were found with EC ZO-1 expressions. Although redox homeostasis is reported to be largely involved in the regulation of tight junction complex in some* in vivo* and* in vitro* studies (Allen and Bayraktutan, 2009; Lochhead et al., 2010), our results showed no significant association. These data collectively suggest that inflammation and leukocyte recruitment but not oxidative stress may be central to HFF diet-induced loss of BBB integrity through the attenuation of tight junction occludin-1 expression. However, it must be noted that the oxidative stress may have been exaggerated during the tissue/cell processing for flow cytometry analysis, which may have masked the effects of HFF feeding.

The data presented provides insight into the potential mechanisms by which pre-diabetes may amplify loss of cognitive function. However, caution must be exercised in interpretation as the manifestation of BBB dysfunction, neuroinflammation and cognitive decline might occur through a lipo-toxic phenomenon rather than pre-diabetes *per se*. Whilst the model chosen in this study is physiologically relevant to a significant proportion of human patients with pre-diabetic insulin resistance, consistent findings in genetically susceptible animal models of T2DM would support the suggested interpretation indicated in this study. Furthermore, a number of studies suggest direct effects of high-fat feeding on hypothalamic fatty acid concentrations and mitochondrial dysfunction, resulting in energy behavior alterations (Sears and Perry, 2015). However, our previous studies consistently observed increased cortical and hippocampal neuroinflammation in high fat fed mice without significant changes in plasma fatty acids (Takechi et al., 2013a,c). Furthermore, a detailed lipidomics analysis on brain lipidome of high fat fed mice revealed no significant changes in lipid homeostasis of cortex and hippocampus compared to low fat fed mice, indicating limited direct impact of high fat feeding on cortical and hippocampal fatty acid concentrations (Giles et al., 2016). Together with our correlation analyses showing significant associations between the BBB permeability and neurodegeneration/cognitive decline, these data collectively suggest the plausible mechanistic involvement of BBB dysfunction in insulin resistance-induced cognitive deficits.

The findings of this study are potentially therapeutically relevant in the context of supporting cognitive function in diabetic insulin resistance. Pharmacological and nutraceutical agents with potent suppressive effects on inflammation and oxidative stress are reported to preserve BBB integrity and prevent neuroinflammation (Pallebage-Gamarallage et al., 2016). A study by Lv et al. (2010) demonstrated that the inhibition of TNF-α by the injection of TNF-α antibodies restored BBB integrity through increased occludin-1 expression. In addition, antibodies of IL-1β were reported to suppress the loss of BBB integrity in human brain microvascular ECs (Didier et al., 2003). Future studies may investigate the efficacy of these agents in preventing BBB disruption and cognitive decline in T2DM. In addition, we note that while the MWM data coupled with the measures of neurodegeneration and astrocyte activation provide robust evidence of cognitive decline, further studies exploring longitudinal changes in BBB integrity with the evolution of frank diabetes and neuropsychobiological measures would be highly informative.

Our results consistently showed that BBB dysfunction precedes the evident neurodegeneration and cognitive decline in HFF-induced pre-diabetic mice. In addition, BBB dysfunction significantly correlated with neurodegeneration and cognitive deficits. Further cellular and molecular investigations suggested that increased inflammation and leukocyte infiltration are involved in the loss of BBB integrity. The data contributes to the understanding of mechanisms by which diabetic insulin resistance accelerates cognitive decline and increases dementia risk and may provide significant therapeutic opportunities.

## Author Contributions

The study was designed and managed by RT and JCLM. The data and samples were collected by RT, VL, CG, NF, SHC, MN and AM, and the samples were analyzed by RT, VL, CG and AM. The data interpretation was done by RT, VL, HA-S and JCLM. The manuscript was prepared and written by RT, VL, EB, CG, NF, AM, SHC, HA-S, MN and JCLM, and the final version was approved by all authors.

## Conflict of Interest Statement

The authors declare that the research was conducted in the absence of any commercial or financial relationships that could be construed as a potential conflict of interest.
