# Supplementary material for: Blood-Brain Barrier Dysfunction Precedes Cognitive Decline and Neurodegeneration in Diabetic Insulin Resistant Mouse Model: An Implication for Causal Link
Source: Front Aging Neurosci. 2017 Dec 1;9:399. doi: 10.3389/fnagi.2017.00399 (PMC5717019; doi:10.3389/fnagi.2017.00399)

**Supplementary Table 1. Calculated nutritional parameters of low-fat control (LF) and high fat/fructose (HFF) diet (Specialty Feeds)**

| Nutritional Parameters | LF (AIN93M) | HFF (SF14-088) |
| --- | --- | --- |
| Protein | 13.6% | 13.6% |
| Total Fat | 4% | 29.9% |
| Crude Fibre | 4.7% | 4.7% |
| Digestible Energy | 15.1 MJ/Kg | 21 MJ/Kg |

**Supplementary Table 2. Ingredients of low-fat control (LF) and high fat/fructose (HFF) diet**

| Ingredients (g/kg) | LF (AIN93M) | HFF (SF14-088) |
| --- | --- | --- |
| Casein (acid) | 140.0 | 140.0 |
| Sucrose | 100.0 | 100.0 |
| Fructose | 0.0 | 150.0 |
| Lard | 0.0 | 300.0 |
| Cellulose | 50.0 | 50.0 |
| Wheat starch | 472.0 | 57.0 |
| Dextrinised starch | 155.0 | 155.0 |
| DL methionine | 1.8 | 1.8 |
| Calcium carbonate | 13.1 | 13.1 |
| Sodium chloride | 2.6 | 2.6 |
| AIN93 trace minerals | 1.4 | 1.4 |
| Potassium citrate | 1.0 | 1.0 |
| Potassium dihydrogen phosphate | 8.8 | 8.8 |
| Potassium sulphate | 1.6 | 1.6 |
| Choline chloride (75%) | 2.5 | 2.5 |
| AIN93 vitamins | 10.0 | 10.0 |
| Cholesterol | 0.0 | 5.0 |

**Supplementary Figure S1. Representative gating strategy for flow cytometry**

The expressions of interleukin-1β (IL-1β), tumour necrosis factor-α (TNF-α), intercellular adhesion molecule-1 (ICAM-1), and reactive oxygen species measured by dihydroethidium (DHE) in the endothelial cells (EC) of cerebrovasculature were determined by flow cytometry in mice maintained on low fat (LF) control diet, high fat/fructose (HFF) diet, HFF with candesartan (Cand) and HFF with ursodeoxycholic acid (UDCA). The population of BBB endothelial cells were identified by gating out CD31^pos^CD45^neg^ cells as shown in the example gating strategy.


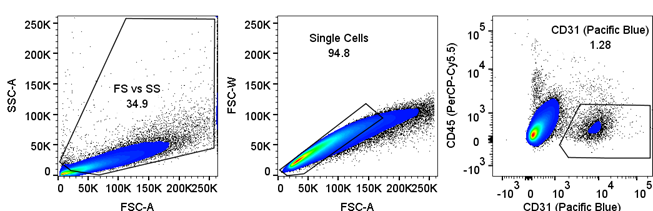


**Supplementary Figure S2. Maze swim speed.**

Mean swim speed during the Morris Water Maze is shown for mice maintained on low fat control chow (LF), high fat and fructose diet (HFF).


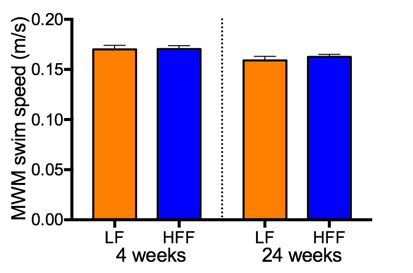

Supplement: Supplementary file 1 [file Data_Sheet_1.docx]
